# *miR-203* Inhibits Frizzled-2 Expression via CD82/KAI1 Expression in Human Lung Carcinoma Cells

**DOI:** 10.1371/journal.pone.0131350

**Published:** 2015-07-01

**Authors:** Mariko Mine, Kojiro Yamaguchi, Tsuyoshi Sugiura, Satomi Chigita, Naoya Yoshihama, Rumi Yoshihama, Naomi Hiyake, Yosuke Kobayashi, Yoshihide Mori

**Affiliations:** 1 Division of Maxillofacial Diagnostic and Surgical Sciences, Department of Oral and Maxillofacial Surgery, Graduate School of Dental Science, Kyushu University Maidashi, Higashi-ku, Fukuoka, Japan; 2 Department of Maxillofacial Diagnostic and Surgical Science, Field of Oral and Maxillofacial Rehabilitation, Graduate School of Dental Science, Kagoshima University Sakuragaoka, Kagoshima, Japan; University of Barcelona, SPAIN

## Abstract

CD82/KAI1, a member of the tetraspanin superfamily, is a suppressor of metastasis and CD82 inhibits canonical Wnt signaling via downregulation of several Frizzled (FZD) isoforms, resulting in accumulation of β-catenin at the cell membrane. In this study, we investigated the mechanism through which CD82 inhibited FZD expression by examining the effects of microRNAs (miRNAs). The miRanda algorithm predicted 11 miRNAs from FZD sequences. Among these miRNAs, CD82 caused upregulation of *miR-203* (by 2.095-fold) and downregulation of *miR-338-3p* (by 0.354-fold) as compared with control cells. Transfection with *miR-203* and *miR338-3p* mimics or inhibitors revealed that *miR-203* downregulated FZD2 mRNA (by 0.268-fold) and protein expression (by 0.701-fold). Moreover, transfection with the *miR-203* mimic also inhibited cell migration. Therefore, these findings suggested that CD82 enhanced the expression of *miR-203* and directly downregulate FZD2 expression, suppressing cancer metastasis by inhibition of the Wnt signaling pathway.

## Introduction

Cancer metastasis is the most crucial event affecting patient prognosis. This multistep event involves the migration of cancer cells from the primary site, avoidance from host defense systems, and subsequent growth of cancer cells at secondary sites. These malignant characteristics of cancer cells are controlled by a complex network of cancer-specific signaling pathways. Thus, elucidation of the signaling mechanisms controlling cancer cell migration and malignant characteristics will provide important insights into the development of chemotherapeutic agents to improve prognoses.

Among the pathways involved in metastatic signaling, the Wnt signaling pathway is a primary target. The first step in this pathway is the binding of secreted Wnt ligands to their receptor, Frizzled (FZD), a seven-pass transmembrane-type receptor with 10 isoforms (FZD1–FZD10) in humans. Binding of Wnt ligands to the FZD-LRP5/6 complex leads the phosphorylation of LRP6 and recruits the Axin-related protein complex. This protein complex then releases β-catenin, which acts as an effector of the canonical Wnt signaling pathway. Released β-catenin in the cytosol accumulates and then transduces the Wnt signal to the nucleus thorough the TCF/LEF complex. In contrast, the noncanonical pathway, which is independent of β-catenin, proceeds through Wnt/planar cell polarity (PCP) and Wnt/protein kinase C (PKC). Both of these Wnt signal pathways are crucial for embryonic development and cancer progression [1]. For example, the expression of FZD1 and -2 is correlated with cancer malignancy and prognosis in breast cancer [2] and colon cancer [3]. However, despite the importance of the biological functions of these proteins, little is known about the gene regulatory mechanisms of Wnts and FZDs.

CD82/KAI1, a member of the tetraspanin superfamily, was originally recognized as a T-cell activation accessory molecule [4]. CD82 is also known to suppress metastasis during cancer progression [5]. Tetraspanins associate with cell surface receptors or proteins and modulate their functions. Moreover, our previous studies revealed a novel function for CD82 in E-cadherin-mediated cellular adhesion [6]. CD82 inhibitsβ-catenin tyrosine phosphorylation and increases the accumulation of E-cadherin/β-catenin complexes at the cell membrane by stabilization of the complex. This function strengthens hemophilic cancer cell adhesion in the primary cancer nest and inhibits invasion and metastasis. We have also revealed that CD82 attenuates Wnt signaling by downregulation of FZD2, -3, -5, -7, and -9 expression without regulating Wnt orβ-catenin expression, leading to inhibition ofβ-catenin nuclear translocation [7].

MicroRNAs (miRNAs) are a class of small noncoding RNAs (~22 nucleotides) that play important roles in the regulation of gene expression. miRNAs induce gene silencing by binding to target sites found within the 3′-untranslated region (UTR) of the target mRNA. This gene silencing leads to inhibition of protein production by suppressing protein synthesis and/or by mRNA degradation. Nearly 50% of human miRNAs are located in cancer-associated genomic regions or in fragile sites [8]. In many types of human cancer, miRNAs behave as oncogenes or anti-oncogenes, termed called onco-miRs and anti-onco-miRs, respectively [9–11]. However, whether miRNAs regulate the Wnt signaling pathway in lung cancer is not yet known.

In this study, we examined the roles of miRNAs in the regulation of FZD expression through CD82 in the Wnt signaling pathway.

## Materials and Methods

### Antibodies

Mouse monoclonal antibodies against CD82 [TS82b] were purchased from Abcam (Cambridge, UK). The anti-FZD antibodies used in this study were as follows: rabbit polyclonal antibodies against FZD1, FZD3, FZD6, FZD8, FZD9, and FZD10; goat polyclonal antibodies against FZD2 (GeneTex, Inc., Irvine, CA, USA); and rabbit polyclonal antibodies against FZD4, FZD5, and FZD7 (Millipore, Temecula, CA, USA).

### Cell culture

The human cell line h1299 (a non-small cell lung carcinoma cell line) and its transfectant derivatives (h1299/zeo and h1299/CD82) were established in our laboratory by transfection of a control vector or *CD82* cDNA and cell sorting-based clone selection techniques, as described previously [12]. h1299/zeo was a mock transfectants cell line exhibiting weak CD82 expression, while h1299/CD82 overexpressed CD82. The protein levels of CD82 in h1299/CD82 cells, as assessed by immunoblotting, were 20 times higher than that in wild-type and h1299/zeo cells, and its cell surface expression, as assessed by flow cytometry, was approximately 9-fold higher than that in wild-type and h1299/zeo cells. The cell lines used in this study were maintained in Dulbecco’s modified Eagle’s medium (DMEM; Sigma, St. Louis, MO, USA) supplemented with 10% fetal bovine serum (FBS; ICN Biomedicals, Aurora, OH, USA) and 2 mM l-glutamine at 37°C and in an atmosphere of 5% CO_2_.

### Transfection with short hairpin RNA (shRNA)

h1299/CD82-sh.control and h1299/CD82-sh.CD82 cell lines were generated by transfection of h1299/CD82 cells with pLKO.1-puro Control Vector (Sigma) and pLKO.1-puro/sh.CD82 (NM_002231; Sigma), respectively, using Lipofectamine (Invitrogen Life Technologies, Carlsbad, CA, USA). Transfected cells were selected by resistance to puromycin (Sigma) and pooled from the individual transfection. The expression levels of CD82 in shRNA-transfected h1299 cells were monitored by reverse transcription-polymerase chain reaction (RT-PCR) and immunoblotting. h1299/CD82-sh.control and h1299/CD82-sh.CD82 cells were maintained in DMEM containing 10% FBS and 2 μg/mL puromycin.

### Transfection with miRNA mimics or miRNA hairpin inhibitors

h1299/zeo and h1299/CD82 cells were seeded at 2 x 10^5^ cells per well in 6-well plates and transfected with miRIDIAN microRNA Mimics, miRIDIAN microRNA Hairpin Inhibitors, or controls (Thermo Fisher Scientific Inc., Waltham, MA, USA). All miRNA mimics, miRNA hairpin inhibitors, and controls were transiently transfected into cells using Lipofectamine RNAiMAX (Invitrogen) according to the manufacturer’s instructions.

### Prediction of possible miRNAs using a web-based application

We searched for miRNAs that potentially targeted FZDs and Wnts using a web-based prediction application, miRanda (http://www.microrna.org; released in August 2010) [13,14]. This program is a database search engine that is commonly used to search for target sites for miRNAs. The algorithm detects partial complementary base sequences. We first searched for miRNAs that would be expected to target FZD2, -3, -5, -7, and -9. From the possible target miRNAs, we excluded miRNAs that targeted other FZD isoforms and Wnts.

### Real-time RT-PCR of mRNAs

Total RNA was extracted from h1299 cells using TRIzol reagent (Invitrogen) and used for first-strand cDNA synthesis. The mRNA levels were quantified in triplicate using a real-time PCR system with a LightCycler FastStart DNA Master SYBER Green 1 kit (Roche Diagnostics, Mannheim, Germany). Specific primers for FZDs were as follows: FZD1 (F: 5′-AGGCTCACCAACAGCAAAC-3′ and R: 5′-TCAGGATTGGCACGAACTC-3′), FZD2 (F: 5′-TGGTTCCATGTTCTTCTCACAG-3′ and R: 5′-ATGTAGGCCACCGCACCAT-3′), FZD3 (F: 5′-AAAGTGAGCAGCTACCACGG-3′ and R: 5′-CCTGGAGTGATCTGTTAGTCG-3′), FZD4 (F: 5′-AGCTGACAACTTTCACACC-3′ and R: 5′-AATGGGGATGTTGATCTTC-3′), FZD5 (F: 5′-CCTAAGGTTGGCGTTGTAATG-3′ and R: 5′-ACAACTTCCCAGTCACAGCA-3′), FZD6 (F: 5′-TCGCCAGCAGCATCCATCT-3′ and R: 5′-TGCCAGGCCAGTGTCAGTAA-3′), FZD7 (F: 5′,GTTTCCCGTTGGTTGTTA-3′ and R: 5′-TTCCTTTAGCGAAGTCAGAA-3′), FZD8 (F: 5′-GCATTGAAGCCTCCCAGAC-3′ and R: 5′-GCTCCAAATCTCGGGTTCT-3′), FZD9 (F: 5′-GCTGTCAAGGTCAGGCAAGT-3′ and R: 5′-CCCTCCACATCCTCCACTA-3′), and FZD10 (F: 5′-CAGTGGATTTGGAGTTGCTTA-3′ and R: 5′-GCACATCGTTTGAGTTCACA-3′).

The PCR cycling conditions were 10 min at 95°C for 1 cycle, followed by 45 cycles at 95°C for 30 s, 60°C for 30 s, and 72°C for 60 s. Amplicons are confirmed that signals are unique by dissociation curve analyses. Expression levels were normalized to theβ*-actin* mRNA level of each sample, as obtained from parallel assays.

### Real-time RT-PCR of miRNAs

For miRNAs, a miRNeasy Mini Kit (Qiagen, Chatsworth, CA, USA) and miScript II RT Kit (Qiagen) were used. The miRNA levels were quantified in triplicate using a real-time PCR system with an miScript SYBR Green PCR kit (Qiagen). The primers for miRNAs (miScript Primer Assays; Qiagen) were as follows: Hs_miR-27a_1 (MS00003241), Hs_miR-27b_2 (MS00031668), Hs_miR-145_1 (MS00003528), Hs_miR-185_1 (MS00003647), Hs_miR-197_2 (MS00008967), Hs_miR-203_1 (MS00003766), Hs_miR-221_1 (MS00003857), Hs_miR-222_2 (MS00007609), Hs_miR-338_1 (MS00003990), Hs_miR-376a_1 (MS00007392), Hs_miR-376b_1 (MS00007399), and Hs_RNU6B_13 (MS00014000).

The PCR cycling conditions were 15 min at 95°C for 1 cycle, followed by 45 cycles at 94°C for 15 s, 55°C for 30 s, and 70°C for 30 s. Amplicons are confirmed that signals are unique by dissociation curve analyses. *RNU6B* was used as miRNA endogenous control.

### Target inhibition analysis of miRNA

We predicted the target site of *miR-203* in the 3′UTR of *FZD2* mRNA by miRanda software, and the specific complimentary sequence for the target site was synthesized using miScript Target Protector (Qiagen). An *miR-203* mimic was transfected into h1299 cells with various concentrations of target protector according to the manufacturer’s instructions. At 48 h after transfection, *FZD2* mRNA levels were measured by real-time PCR.

### Immunoblot analysis

Cell lysates for immunoblotting were prepared in cell lysis buffer (1% Triton X-100, 150 mM NaCl, 0.5% sodium deoxycholate, 0.1% sodium dodecyl sulfate [SDS], and 50 mM Tris-HCl [pH 8.0]). The samples were resolved by SDS-polyacrylamide gel electrophoresis (PAGE), transferred to nitrocellulose membranes (Bio-Rad, Hercules, CA, USA), and incubated with specific primary antibodies. Protein bands were visualized using horseradish peroxidase (HRP)-conjugated secondary antibodies and Enhanced Chemiluminescence Reagent (Amersham Pharmacia Biotech, Piscataway, NJ, USA). Densitometric analyses were performed using computer-assisted densitometry (ChemiDoc XRS-J; Bio-Rad) and Quantity One software (Bio-Rad).

### Wound healing assay

h1299/zeo and h1299/CD82 cells were seeded at 2 × 10^5^ cells per well in 6-well plates and transfected with mimic miRNAs, hairpin inhibitor miRNAs, or controls. At 48 h after transfection, wounds were created by scratching with a 200-μL pipette tip. Cell monolayers were then washed with medium, and wounds were observed under a fluorescence microscope (BZ-8000; Keyence, Osaka, Japan). The wound regions were photographed again after 8, 16, or 24 h in order to measure the wound area. Wound area was standardized by using the following formula: wound area (% of control) = wound area after the indicated period × 100 / initial wound area. All experiments were carried out in triplicate and repeated three times.

### Statistical analysis

Data are shown as means ± SDs. All data were analyzed via Student’s *t*-test in SPSS 13.0 software. Differences with *P* values of less than 0.05 were considered significant.

## Results

### Prediction of miRNAs that potentially targeted FZD2, -3, -5, -7, and -9

We have previously reported that CD82 downregulates the expression of FZD2, -3, -5, -7, and -9 but does not regulate Wnt or other FZD isoforms [7]. Therefore, to identify miRNAs that downregulate only FZD2, -3, -5, -7, and -9, we first predicted common miRNAs targeting FZD2, -3, -5, -7, and -9 using miRanda [13]. We excluded miRNAs that also targeted other FZD isoforms and Wnts from the predicted miRNAs. As a result, we selected 11 miRNAs (*miR-27a*, *miR-27b*, *miR-145*, *miR-185*, *miR-197*, *miR-203*, *miR-221*, *miR-222*, *miR-338-3p*, *miR-376a*, and *miR-376b*) that were potentially regulated by CD82 to target FZD2, -3, -5, -7, and -9 for further study (Table 1). The mirSVR scores of these miRNAs, which indicate the possibility of their downregulation at the mRNA level [14], are also shown (Table 1).

**Table 1:** Predicted miRNAs and target FZDs.

miRNA	Target genes (mirSVR score)
**27a**	FZD5 (-0.1505), FZD7 (-0.4657)
**27b**	FZD5 (-0.1505), FZD7 (-0.4657)
**145**	FZD6 (-0.1497), FZD7 (-0.1317), FZD9 (-0.1073)
**185**	FZD7 (-0.1232) (-0.7958)
**197**	FZD3 (-0.8602) (-1.1091)
**203**	FZD1 (-0.3899) (-0.5238), FZD2 (-0.9949), FZD3 (-0.2282), FZD4 (-0.1226), FZD5 (-0.1469), FZD6 (-0.6731)
**221**	FZD3 (-0.2186) (-0.1455)
**222**	FZD3 (-0.1455)
**338-3p**	FZD3 (-1.2252), FZD7 (-0.2038)
**376a**	FZD3 (-1.0569), FZD5 (-0.4393)
**376b**	FZD3 (-1.0569), FZD5 (-0.4393)

### Expression levels of the 11 selected miRNAs in h1299 cells

**Fig 1:**
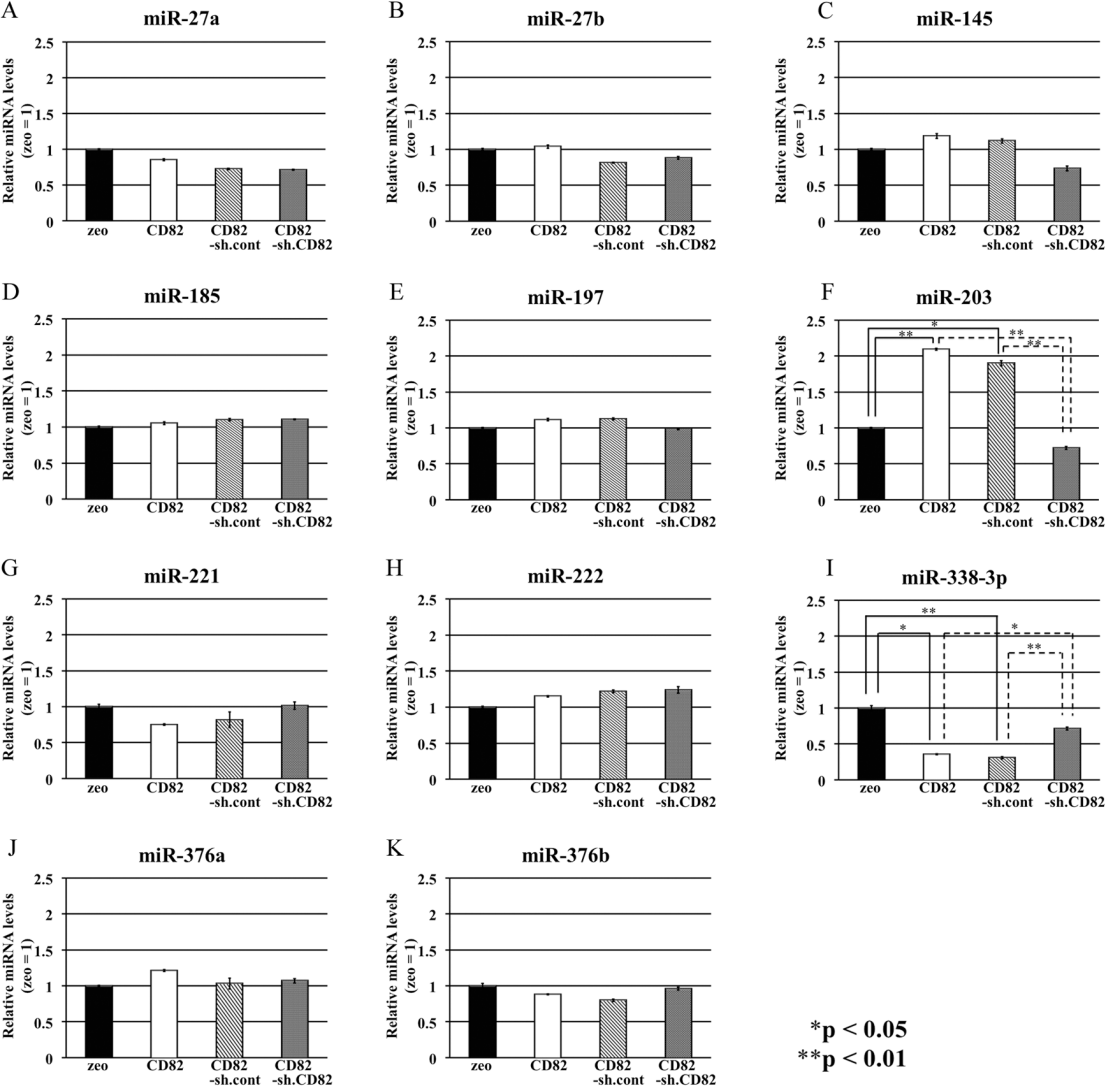
Expression of 11 miRNAs in h1299 cells. Total RNA was isolated from h1299 cells, and the 11 predicted miRNA levels were analyzed by real-time RT-PCR. *RNU6B* was used as an internal reference gene. Experiments were performed in triplicate, and relative miRNA levels (zeo = 1) were averaged. Asterisks indicate statistically significant differences (*p < 0.05, **p < 0.01) between the two values. Data are presented as the means ± SDs.

Next, we used real-time PCR to confirm the expression of the 11 selected miRNAs in h1299 cells. *miR-338-3p* was significantly downregulated in h1299/CD82 cells (0.354-fold), whereas *miR-203* was significantly upregulated (2.095-fold), as compared with h1299/zeo cells. Additionally, knockdown of CD82 by shRNA in h1299/CD82 cells allowed recovery of miRNA levels to those in h1299/zeo cells. These results suggested that CD82 specifically regulated these miRNAs. No significant differences in *miR-27a*, *miR-27b*, *miR-145*, *miR-185*, *miR-197*, *miR-221*, *miR-222*, *miR-376a*, or *miR-376b* expression levels were observed between h1299/zeo and h1299/CD82 cells (Fig 1).

### Effects of *miR-338-3p* and *miR-203* on the expression levels of *FZD* mRNAs in h1299 cells

**Fig 2:**
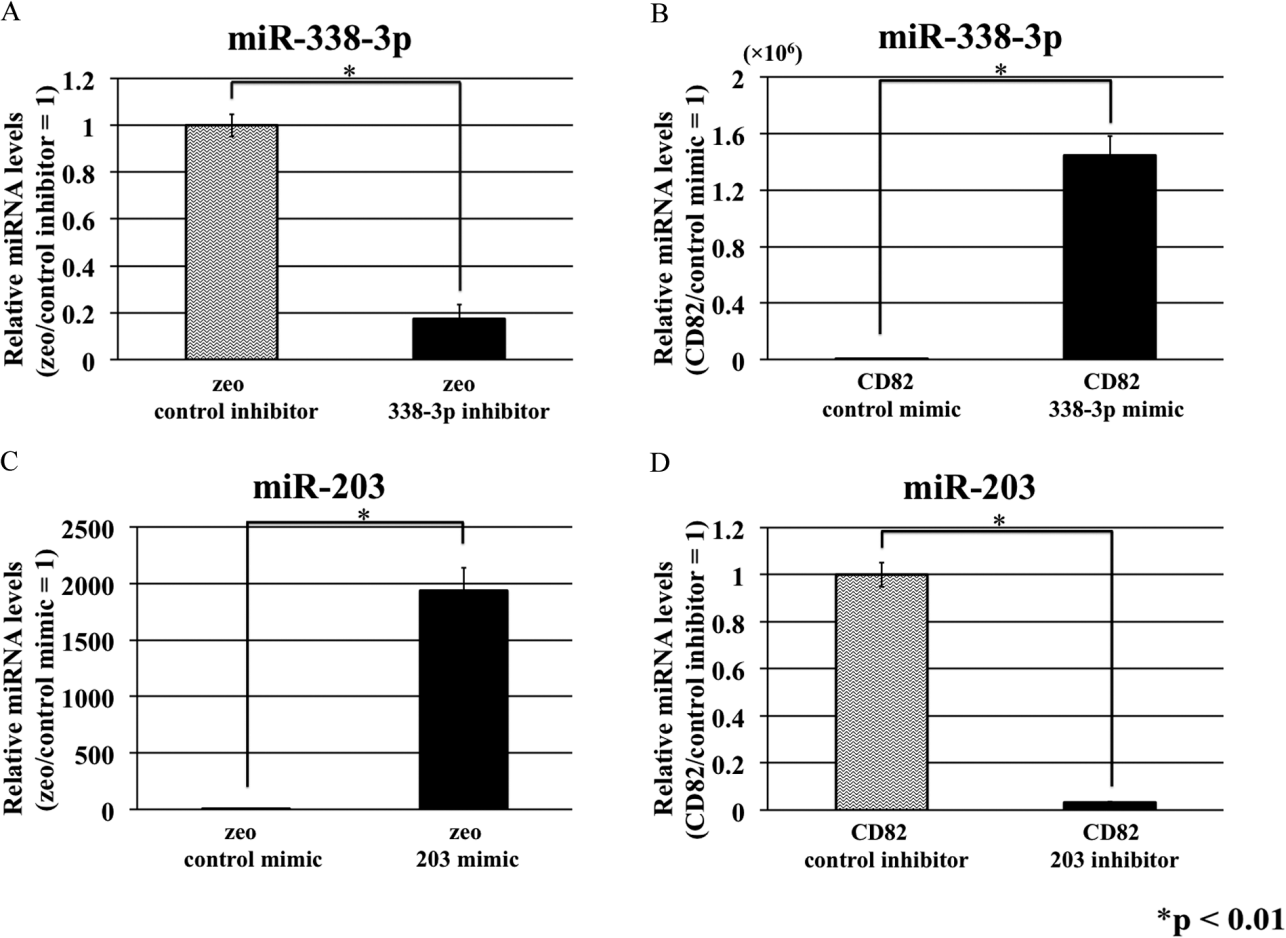
Transfection with miRNA mimics and hairpin inhibitors. Total RNA was isolated from h1299 cells at 48 h after transfection with miRNA mimics or miRNA hairpin inhibitors, and miRNA levels were then analyzed by real-time RT-PCR. *RNU6B* was used as an internal reference gene. Experiments were performed in triplicate, and relative miRNA levels (zeo/control inhibitor = 1) were averaged. The asterisks in the figure indicate statistically significant differences (*p < 0.01) between the two values. Data are presented as the means ± SDs.

To examine the functional effects of *miR-338-3p* and *miR-203* on the regulation of FZDs, we transiently transfected h1299/zeo and h1299/CD82 cells with *miR-338-3p* or *miR-203* mimics or inhibitors. Forty-eight hours after transfection, miRNA levels of *miR-338-3p* and *miR-203* were significantly decreased in h1299/zeo cells transfected with the *miR-338-3p* inhibitor and h1299/CD82 cells transfected with the *miR-203* inhibitor (Fig 2A and 2D). Transfection with *miR-338-3p* and *miR-203* mimics significantly increased the miRNA levels in h1299/CD82 cells transfected with *miR-338-3p* mimic and in h1299/zeo cells transfected with the *miR-203* mimic (Fig 2B and 2C).

**Fig 3:**
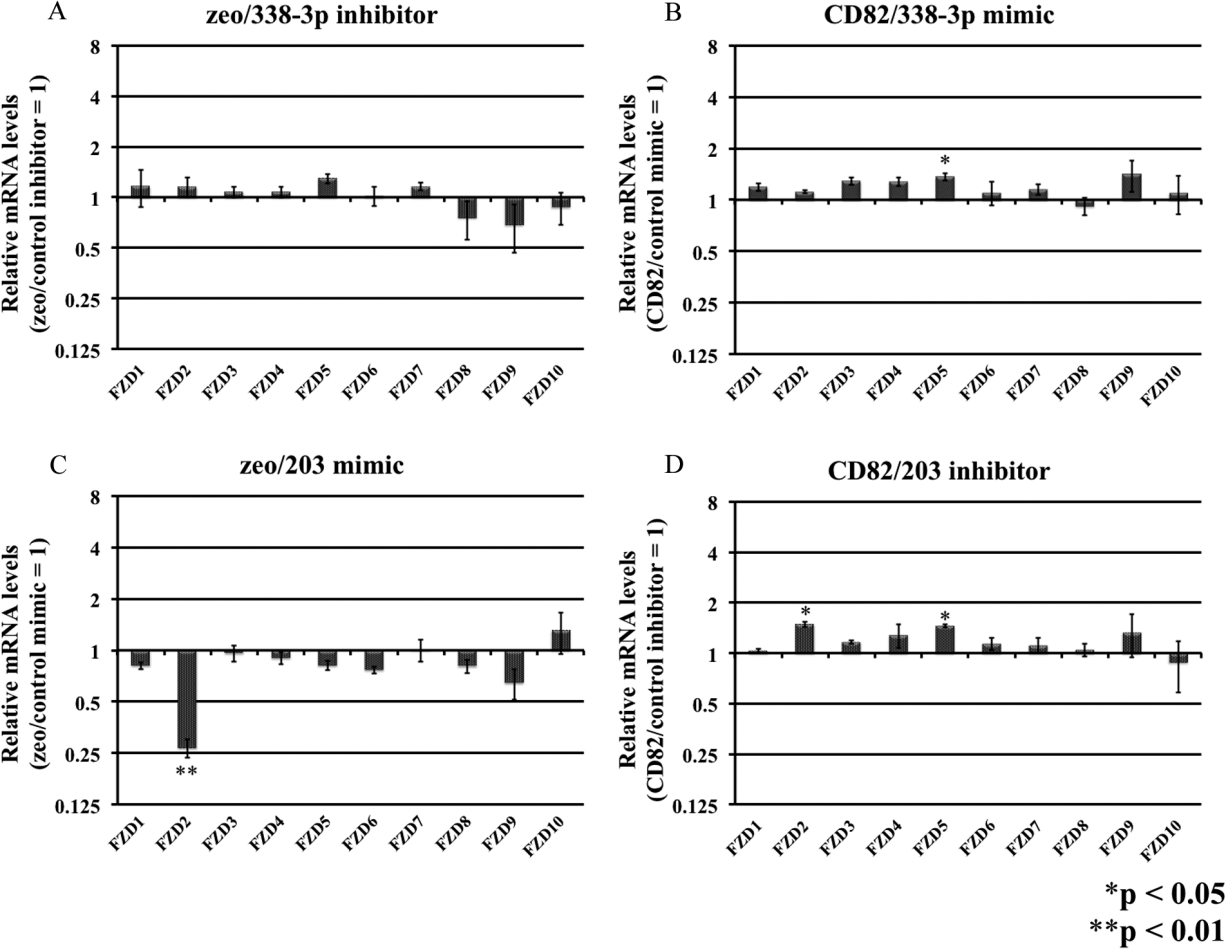
Effects of *miR-338-3p* and *miR-203* on the expression of *FZD* mRNA. h1299 cells were transfected with miRNA mimics or miRNA hairpin inhibitors, and total RNA was isolated after 48 h. mRNA levels were then analyzed by real-time RT-PCR. β-Actin was used as an internal reference gene. Experiments were performed in triplicate, and relative miRNA levels (zeo/control inhibitor = 1) were averaged. Data are presented as the means ± SDs.

**Fig 4:**
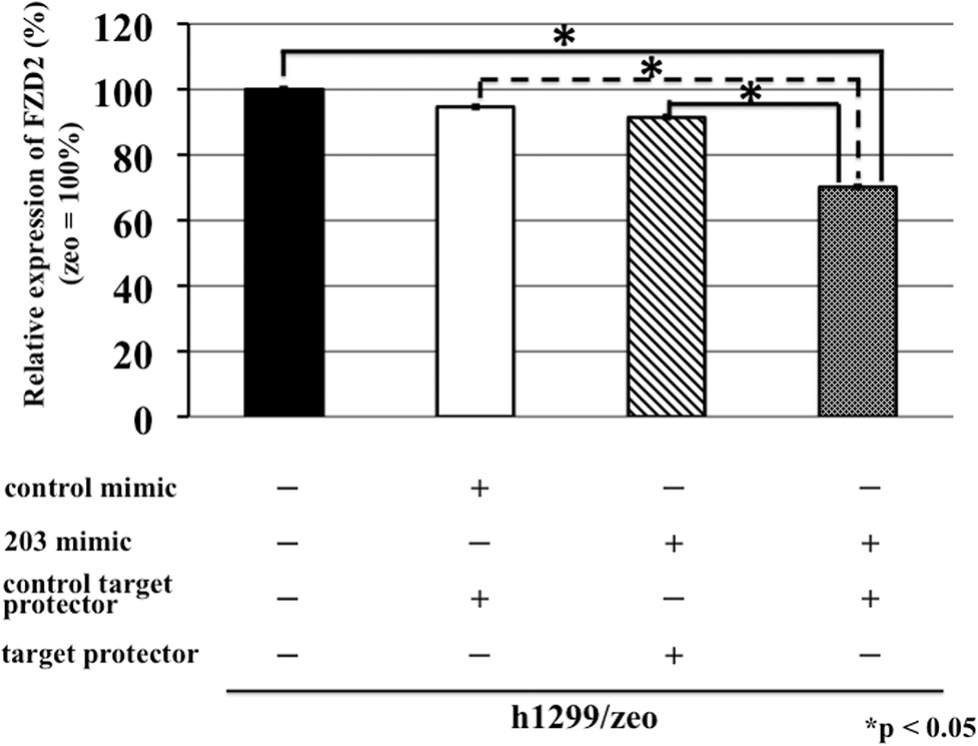
Target inhibition assay for determination of the effects of *miR-203* on *FZD2* mRNA expression. An *miR-203* mimic was transfected into h1299 cells with or without target protector. At 48 h after transfection, *FZD2* mRNA levels were measured by real-time RT-PCR. β-Actin was used as an internal reference gene. Experiments were performed in triplicate, and relative *FZD2* mRNA (zeo = 100%) were averaged. Data are presented as the means ± SDs.

Next, we examined the expression levels of *FZD* mRNAs in these transfected h1299 cells. In cells transfected with *miR-338-3p* mimic (h1299/CD82/338-3p mimic) or hairpin inhibitor (h1299/zeo/338-3p inhibitor), the mRNA levels of FZDs did not differ compared with control cells (Fig 3A and 3B). In contrast, h1299 cells transfected with *miR-203* mimic showed significant downregulation of *FZD2* mRNA levels compared to control cells (0.268-fold; Fig 3C). To confirm whether *miR-203* targeted FZD2 directly, we perform target inhibition assays (Fig 4). The target protector designed for the *miR-203* target site in *FZD2* mRNA completely negated the inhibitory effects of the *miR-203* mimic and CD82 on *FZD2* mRNA expression.

### Effects of *miR-338-3p* and *miR-203* on the protein levels of FZDs in h1299 cells

**Fig 5:**
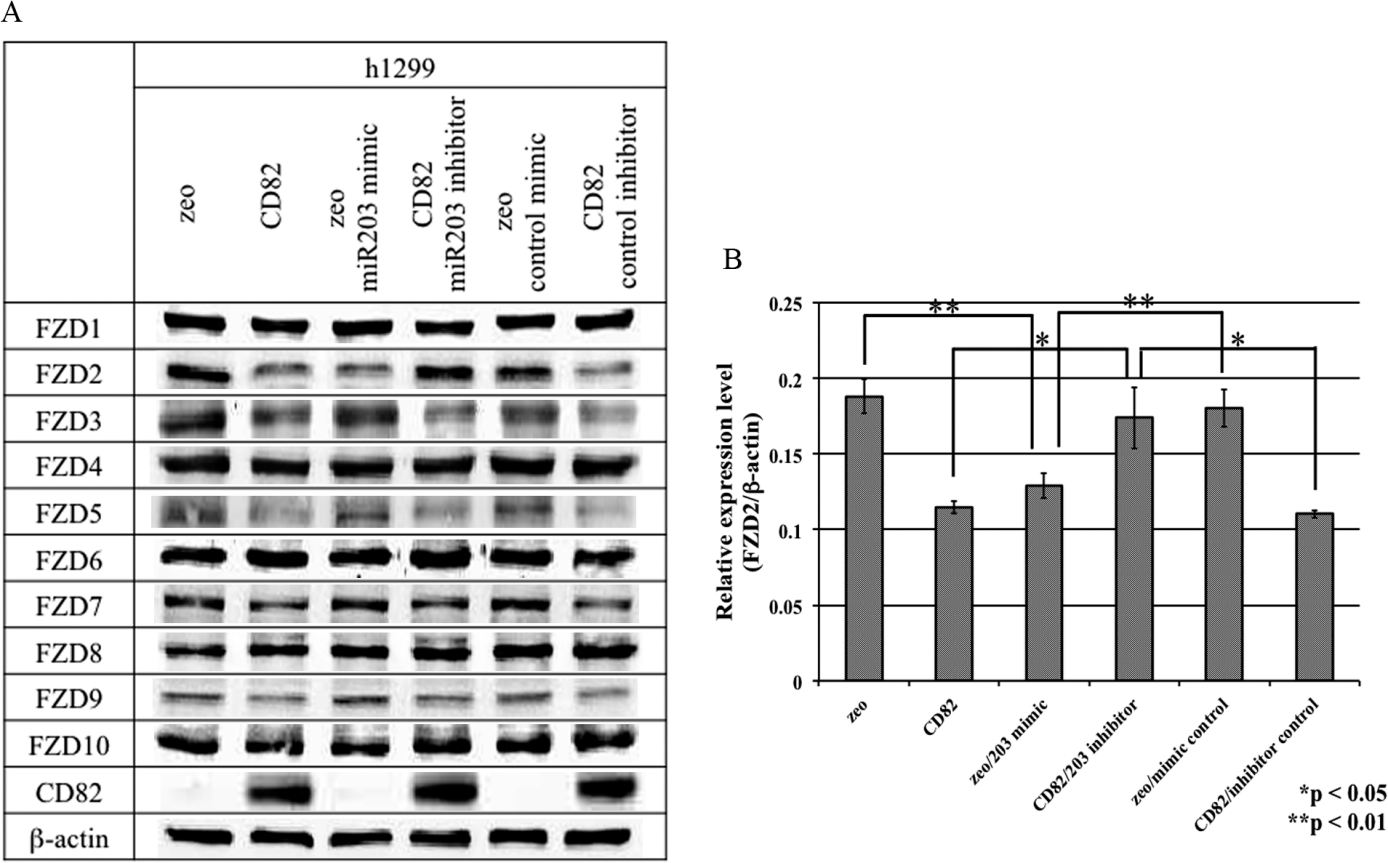
Effects of *miR-203* on the expression of Frizzled (FZD) proteins. h1299 cells (h1299/zeo, h1299/CD82) were transfected with *miR-203* mimic, *miR-203* inhibitor, or miR controls. At 48 h after transfection, total protein was extracted and analyzed by immunoblot analysis with anti-FZD antibodies. The same blots were stripped and reprobed with anti-β-actin antibodies as a loading control. Experiments were repeated three times, and the most representative data are shown (A). A densitometric analysis was performed using the data from (A), followed by normalization to the densitometric value of β-actin. The resulting relative expression values (FZD2/β-actin) are indicated in (B). Data are presented as the means ± SDs. Asterisks indicate statistically significant differences (*p < 0.05, **p < 0.01) between the two values.

Next, we examined the protein levels of FZDs by immunoblot analysis in h1299 cells transfected with miRNA mimics or inhibitors. h1299/zeo cells transfected with the *miR-203* mimic showed significant downregulation of FZD2 protein levels compared to parental and control cells (0.701-fold; Fig 5A and 5B). In contrast, h1299/CD82 cells transfected with the *miR-203* inhibitor showed a significant increase in FZD2 protein levels compared to parental and control cells (1.546-fold; Fig 5A and 5B).

**Fig 6:**
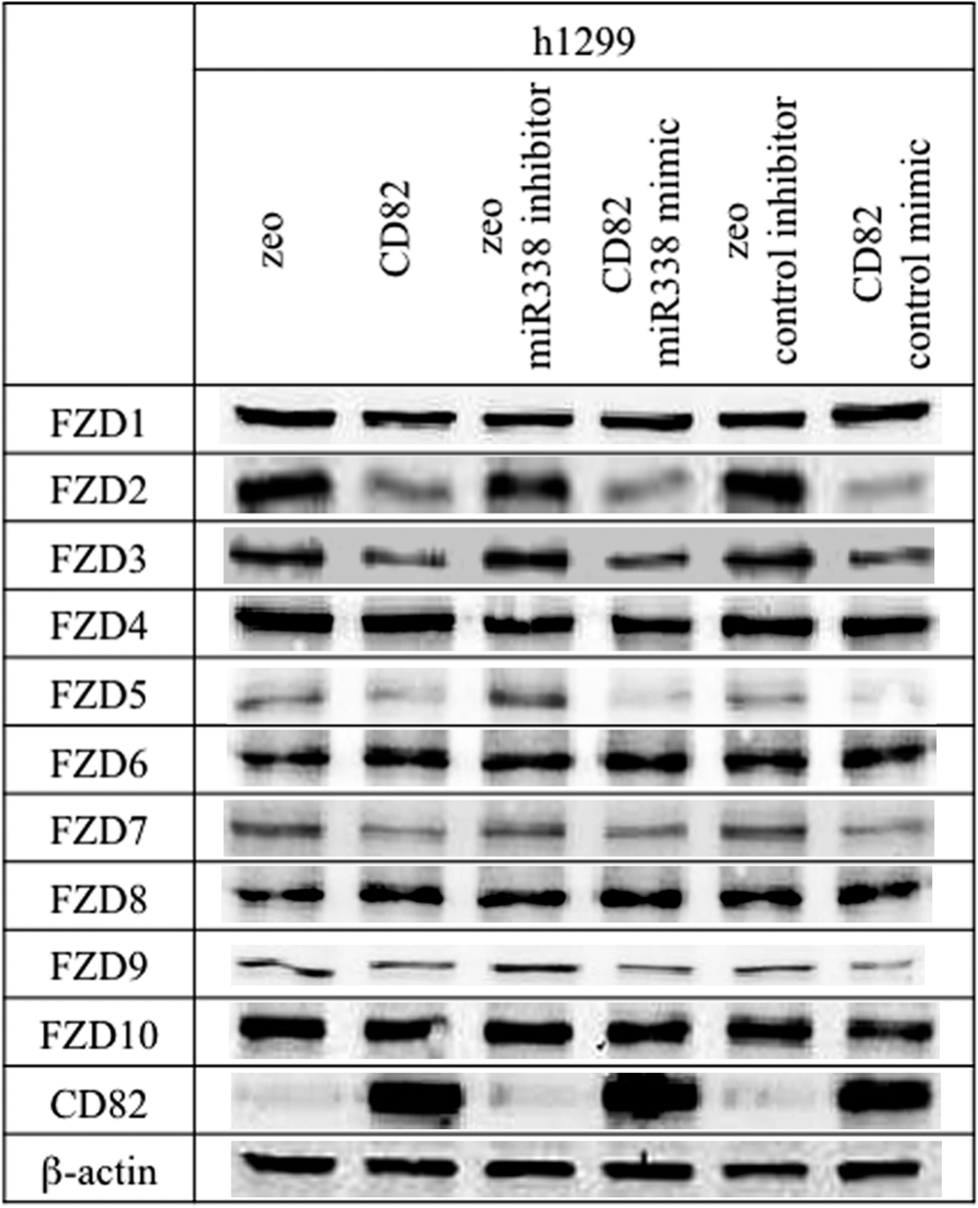
Effects of *miR-338-3p* on the expression of Frizzled (FZD) proteins. h1299 cells (h1299/zeo, h1299/CD82) were transfected with *miR-338-3p* mimic, *miR-338-3p* inhibitor, or miR controls. At 48 h after transfection, total protein was extracted and analyzed by immunoblot analysis with anti-FZD antibodies. The same blots were stripped and reprobed forβ-actin as a loading control. Experiments were repeated three times, and the most representative data are shown.

In contrast, cells transfected with the *miR-338-3p* mimic or inhibitor showed no significant differences compared to parental and control cells (Fig 6).

### Effects of *miR-338-3p* and *miR-203* on the migration ability of h1299 cells

**Fig 7:**
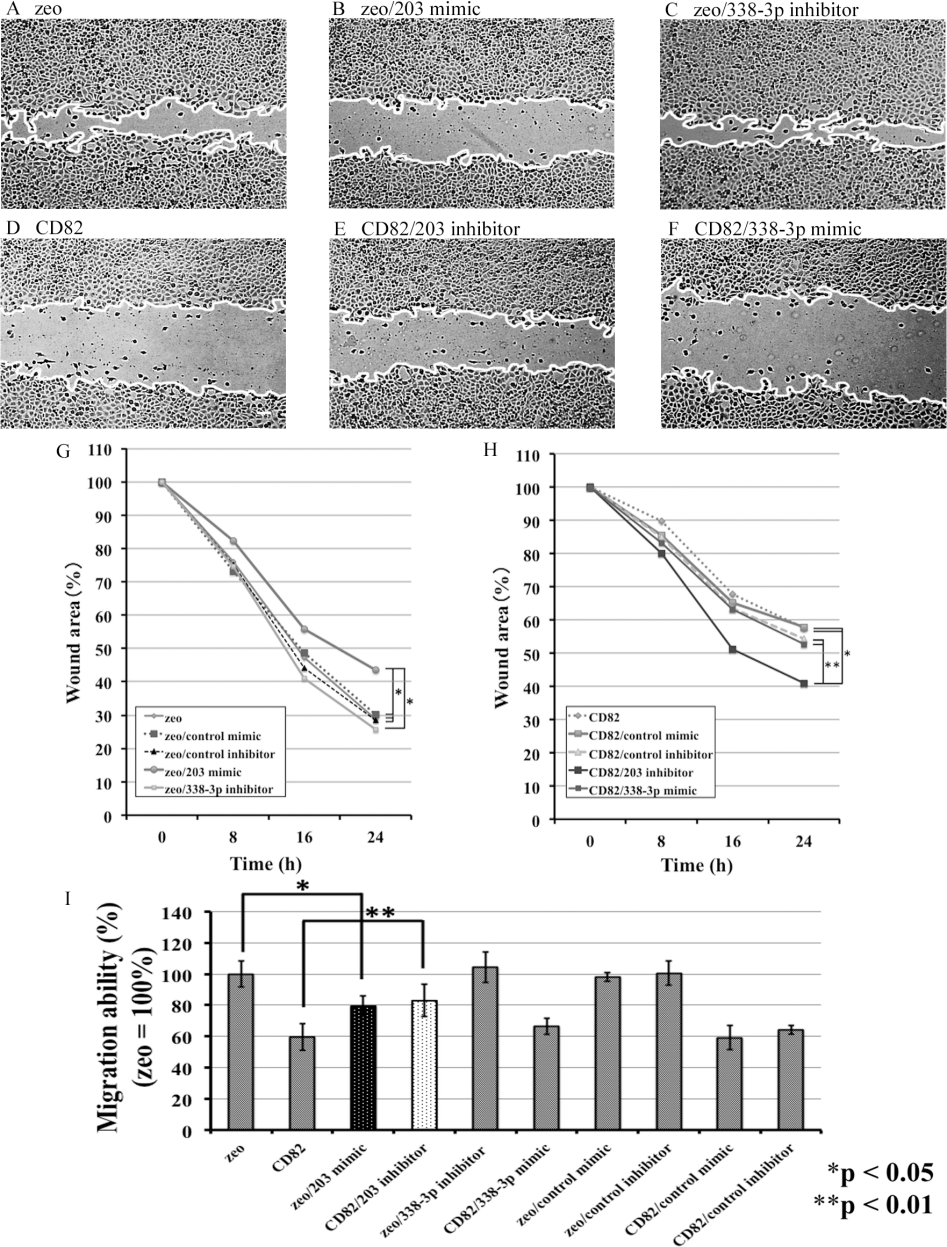
Effects of *miR-203* and *miR-338-3p* transfection on the migration of h1299 cells. (A–F) Cell migration was evaluated using wound healing assays, as described in the Materials and Methods. Randomly chosen wound fields were photographed every 8 h for 24 h. (G, H) Wound areas were evaluated using the following formula: wound area (%) = wound area after the indicated period × 100 / initial wound area. (I) Migration ability was evaluated based on the wound area. The experiments were performed in triplicate, and the data were calculated as means ± SDs. The statistical significance of differences was analyzed using the Student’s t-test. *P < 0.01, **P < 0.05.

To elucidate the functional effects of FZD2 downregulation by *miR-203*, cell migration was quantitatively examined by wound healing assay. The *miR-203* mimic inhibited cell migration in h1299/zeo cells (Fig 7A, 7B and 7G), while the *miR-203* inhibitor induced cell migration in h1299/CD82 cells (Fig 7D, 7E and 7H). In contrast, *miR-338-3p* did not affect h1299 cell migration (Fig 7A, 7C, 7D and 7F). Fig 7I shows the inhibition of migration in h1299 cells. Overexpression of CD82 reduced cell migration to approximately 60% that of h1299/zeo cells. The *miR-203* mimic inhibited migration in h1299/zeo cells, and the *miR-203* inhibitor induced migration in h1299/CD82 cells, reaching approximately 80% that of h1299/zeo cells.

## Discussion

**Fig 8:**
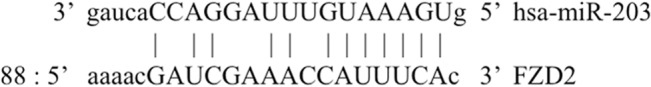
*miR-203* targeted the *FZD2* gene. The FZD2 3′-UTR sequence and complementary *miR-203*-binding sequences are shown.

The mechanisms regulating FZD expression have not been elucidated. Recently, researchers have reported that FZD is regulated by miRNAs [16,17]. Therefore, in this study, we analyzed the effects of miRNAs on FZD expression. Using the miRanda algorithm [13,14], we predicted 11 candidate miRNAs that targeted specific FZDs. Of these, *miR-203* and *mir-338-3p* were regulated by CD82 overexpression. After subsequent analyses, only *miR-203* inhibited FZD2 expression at the mRNA and protein levels. As shown in Fig 8, FZD2 had one potential complimentary *miR-203*-binding site within its 3′-UTR, suggesting that FZD2 was targeted by *miR-203*. Additionally, target inhibition assays designed for this binding site revealed that *miR-203* targeted *FZD2* mRNA directly. Moreover, *miR-203* also regulated cell migration, a critical component of metastatic progression, in cancer cells through CD82. Thus, our data provided important insights into the regulation of the Wnt signaling pathway by miRNAs in cancer cells.

CD82/KAI1 is recognized as an antimetastatic factor and has been reported to attenuate or modify cell membrane receptor signaling, including integrin, epidermal growth factor receptor (EGFR), and c-Met pathways [6,12,15]. We have previously reported that CD82 regulates canonical Wnt signaling by downregulating specific FZDs (FZD2, -3, -5, -7, and -9) [7]. These FZDs are mainly part of the noncanonical Wnt pathway (Wnt/PCP and Wnt/PKC), with the exception of FZD9. Therefore, CD82 inhibits both canonical Wnt signaling and noncanonical Wnt signaling by suppressing FZD receptor expression. Suppression of both Wnt signaling pathways is known to contribute to cancer progression via various mechanisms. Additionally, our analysis demonstrated that miRNAs were involved in the CD82-dependent regulation of FZDs, thereby providing insights into these complex regulatory mechanisms.

*miR-203* has been reported to act as a tumor suppressor in malignant melanoma [18], glioma [19,20], and esophageal squamous cell carcinoma [21], but as a tumor promoter in epithelial ovarian cancer [22]. Various types of *miR-203* target genes have been reported, including versican, an extra cellular matrix proteoglycan [18], phospholipase D2 (PLD2) [20], Robo1/ERK/MMP9 [19], PI3KCA [23], CREB1 [24], LASP1, NUAK1, and SPARC [25]. This is the first report that *miR-203* directly regulated FZD2 mRNA and protein levels. FZD2 is one of the most important receptors in the noncanonical Wnt pathway. In many types of cancer, FZD2 expression is strongly correlated with poor prognosis [2,3,26,27]. In particular, Wnt5a/FZD2 signaling has been shown to control melanoma Ca^2+^ homeostasis [28], cellular migration, and invasion in colon cancer [3]. Interestingly, *miR-203* inhibits cancer stem cell maintenance by self-renewal inhibition through indirect induction of DKK1, a secreted antagonist of the canonical Wnt pathway [29]. Thus, *miR-203* can inhibit both canonical and noncanonical Wnt pathways. This function corresponds to that of CD82 and supports the potential crosstalk between CD82 and *miR-203*.

However, the mechanism through which CD82 downregulates *miR-203* is still unclear. CD82 forms complexes with cell membrane receptors and modifies their signaling pathways. We previously reported that CD82 attenuates EGFR and c-Met signaling by binding to these receptors [6,12,15]. Recently, EGFR and c-Met receptor tyrosine kinase were shown to control miRNA expression [30]. In this previous report, overexpression of *miR-203* in A549 cells was shown to reduce AKT phosphorylation and the expression of its substrate GSK3β. This finding was also consistent with our previous report showing that overexpression of CD82 downregulates GSK3β [7]. Furthermore, c-Met receptor inhibits the expression of *miR-203*, and forced expression of CD82 attenuates c-Met signaling in h1299 cells. These reports suggest that CD82 enhances the expression of *miR-203* via attenuation of c-Met signaling. However, this indirect evidence needs to be confirmed in further studies.

Importantly, in this study, we confirmed the functional effects of *miR-203* transfection in cancer cells. Transfection of h1299/zeo cells led to a 20% reduction in cell migration compared to parental h1299/zeo cells, and this level of reduction was half of that in h1299/CD82 cells (40%). These results suggested that FZD3, -5, and -7, which are also downregulated by CD82 expression, may contribute to CD82 function. These mechanisms should be investigated in further studies.

In conclusion, we found that CD82 enhanced the expression of *miR-203* and directly downregulated FZD2 expression. Accumulating evidence has demonstrated the presence of abnormal *miR-203* expression in cancer stem cell. Therefore, this novel mechanism for the regulation of FZD2 expression via *miR-203* could be utilized for epigenetic therapy for cancer stem cells.
